# Experimental Research on Creep Characteristics of Nansha Soft Soil

**DOI:** 10.1155/2014/968738

**Published:** 2014-01-09

**Authors:** Qingzi Luo, Xiaoping Chen

**Affiliations:** MOE Key Laboratory of Disaster Forecast and Control in Engineering, College of Science and Engineering, Jinan University, Guangzhou 510632, China

## Abstract

A series of tests were performed to investigate the creep characteristics of soil in interactive marine and terrestrial deposit of Pearl River Delta. The secondary consolidation test results show that the influence of consolidation pressure on coefficient of secondary consolidation is conditional, which is decided by the consolidation state. The ratio of coefficient of secondary consolidation and coefficient of compressibility *C*_*a*_/*C*_*c*_ is almost a constant, and the value is 0.03. In the shear-box test, the direct sheer creep failure of soil is mainly controlled by shear stress rather than the accumulation of shear strain. The triaxial creep features are closely associated with the drainage conditions, and consolidation can weaken the effect of creep. When the soft soil has triaxial creep damage, the strain rate will increase sharply.

## 1. Introduction

With the rapid development of the economy in China, infrastructure construction is increasing prosperously, which bring the long-term settlement, long-term strength, and other issues into people's sight. The soft soil is distributed widely in China, and its deformation and strength are time-dependent, so the effect of creep cannot be neglected. Some experimental and theoretical researches have been done by investigators to analyze the creep characteristics quantitatively and qualitatively.

The secondary consolidation phenomenon of soft soil was studied by Taylor and Merchant [1], and they thought of the secondary consolidation settlement of soil caused by the frictional resistance of soil particles. Mesri and Godlewski [2] summarized the results of secondary consolidation of normally consolidated soil obtained by predecessors, and they found a good linear relationship between the secondary consolidation coefficient *C*_*a*_ and compression index *C*_*c*_. Newland and Allely [3] discussed the influence of consolidation pressure on the secondary consolidation coefficient *C*_*a*_, and they considered that *C*_*a*_ has nothing to do with consolidation pressure but upon its preconsolidation pressure. However, Nash et al. did not agree with that viewpoint and reported that *C*_*a*_ increased firstly and then decreased with the increasing of consolidation pressure. Some argument that focused on the relationship between consolidation pressure and *C*_*a*_ were discussed by Leroueil et al. [4]. Secondary consolidation just has bulk creep; however, triaxial creep is the coupling between shear creep and volume creep of soil. Bishop and Lovenbury [5] did experiments to investigate the creep characteristics of undisturbed clays, which were further studied by other researches [6–9].

Soft soil in interactive marine and terrestrial deposit has the common characters of general soft soil, such as higher water content, a greater void ration, higher compressibility, lower shear strength, and higher sensitivity [10]. However, due to its special sedimentary environment and high content of silt, there are some basic physical and mechanical properties different from marine soft soil. In this paper, a series of trials were performed to study the creep characters of soft soil in interactive marine and terrestrial deposit taken from Nansha of Guangzhou. Water contents *w* were 46%–73%, and void ratio *e*_0_ were 1.71–2.16, both of which were of great variability and less than those of marine soft soil. Average liquid limit *W*_*L*_ of the soil samples was around 47.8%, and average plastic limit *W*_*P*_ was about 24%.

## 2. Experiments

### 2.1. Secondary Consolidation Test

When the primary consolidation is completed, the creep deformation of soft soil will continue with the prolonging of time, and the secondary consolidation effect cannot be ignored. The soft soil samples from different depths, which had the approximate water content (about 56%) and density (1.72 g/cm^3^), were investigated in this work, and the secondary consolidation properties of soft soil samples were researched by consolidation test. The specimens with a diameter of 61.8 mm and a height of 20 mm were studied, and the test scheme is as follows.

1. The sampling depth of soft soil sample No. 1 is 6 m. The loading sequence is 12.5 → 25 → 50 → 100 → 200 → 400 kPa, and loading time for each stage of loading is three days.
2. The sampling depth of soft soil sample No. 2 is 16 m. The loading sequence is 50 → 100 → 200 → 400 kPa, and loading time for each stage of loading is three days.

### 2.2. Shear-Box Test

**Figure 1:**
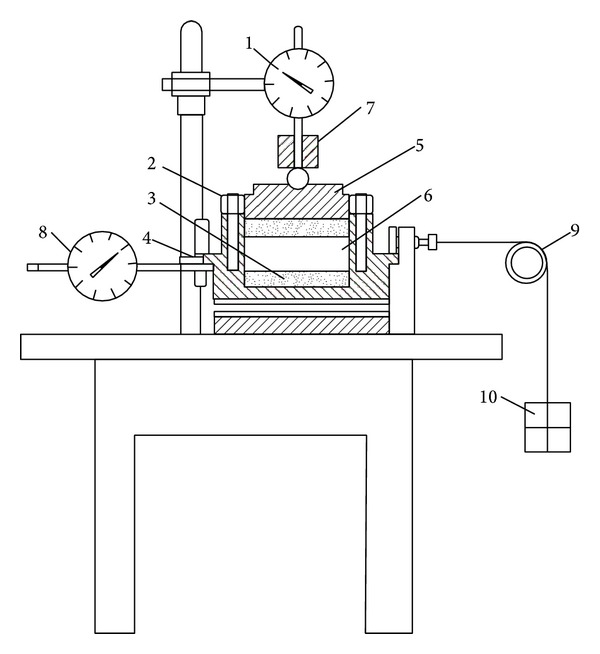
Schematic diagram of shear box. (1) Vertical displacement meter; (2) upper box; (3) permeable stone; (4) lower box; (5) cover plate for loading; (6) specimen; (7) compression framework; (8) horizontal displacement meter; (9) pulley; and (10) weight.

In the shear-box tests, the undisturbed soil specimen had the same size as the specimen in the consolidation test, and the water content was 48%; the density was 1.74 g/cm^3^. Shear-box tests were performed by the direct shear apparatus controlled by the shearing force, which was refitted by the routine strain-controlled direct shear apparatus, to investigate the shear creep characters of soft soil in interactive marine and terrestrial deposit, and schematic diagram is plotted in Figure 1. Each level of shear stresses applied in experiments was determined in the following way: conducting consolidated-drained direct shear tests to determine the peak strength *τ*_*f*_ of soil samples under different consolidation pressures, then according to the formula *τ*_*i*_ = *τ*_*f*_/*n* to define each level of shear stresses in shear creep tests, where *n* is the loading times and the value is 4~6. The peak strengths of consolidated-drained direct shear tests under different consolidation pressures are listed in Table 1, and the duration of each stage of shear stress was determined by the criterion that the shear deformation is smaller than 0.01 mm/day.

### 2.3. Triaxial Creep Test

The mechanism of the triaxial creep is different from the mechanisms of secondary consolidation and shear creep, which is the coupling between shear creep and volume creep of soil. In order to make further study on the creep properties of soft soil in interactive marine and terrestrial deposit, both drained creep test and undrained creep test were performed by stress-controlled triaxial apparatus, and the indoor temperature was maintained at 24°C to eliminate the influence of temperature on the test. In triaxial creep test, the specimen had a diameter of 39.1 mm and a height of 8 mm, whose water content was 54%, and density was 1.68 g/cm^3^. In those tests, soil samples were consolidated for two days under confining pressure of 200 kPa firstly, and then each level of deviatoric stresses were applied by steps under different drainage conditions, which lasted three days. The peak strength *τ*_*f*_  of triaxial test under a certain all-around pressure (*p* = 200 kPa) was obtained by routine triaxial apparatus, which was 210 kPa for drained triaxial test and 100 kPa for undrained triaxial test, used to determine the loading times.

## 3. Results and Discussion

### 3.1. Secondary Consolidation

**Figure 2:**
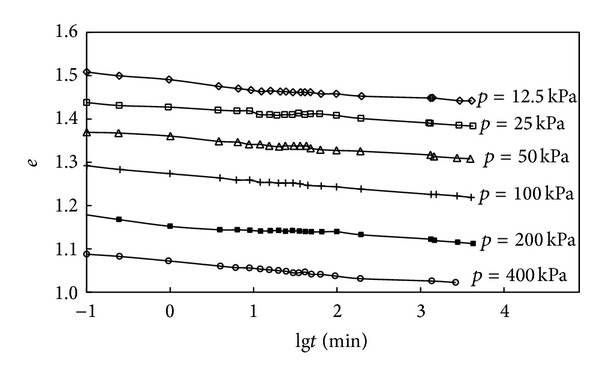
Tested *e*-lg*t* curves of soft soil sample No. 1.

**Figure 3:**
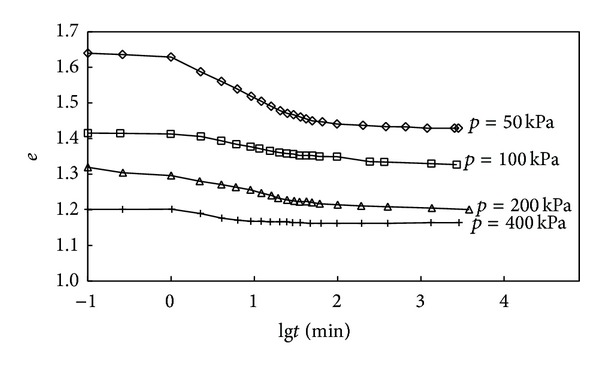
Tested *e*-lg*t* curves of soft soil sample No. 2.

**Figure 4:**
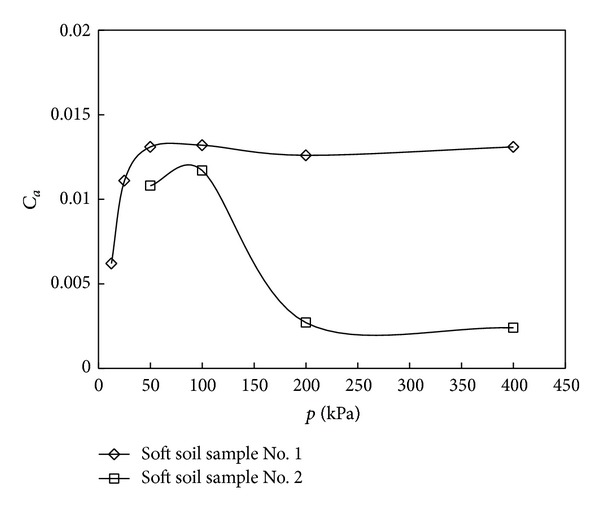
Curves of secondary consolidation coefficient *C*_*a*_ and consolidation load *p*.

The tested *e*-lg*t* curves of undisturbed soil from different depth are given in Figures 2 and 3, and the relationship between coefficient of secondary consolidation and consolidation pressure is shown in Figure 4.

It can be seen from Figure 2 that (1) the partition of primary-secondary consolidation is not obvious; (2) when *p* ≥ 50 kPa, the *e*-lg*t* curves are parallel to each other under each stage of loading, which is to say the coefficient of secondary consolidation does not depend on the consolidation load and stay a constant. But when consolidation load is small, the coefficient of secondary consolidation has some changes. Those conclusions also can be got from Figure 3, and we can find, when the *p* ≥ 50 kPa, the coefficient of secondary consolidation of soft soil sample No. 1 comes to a smooth stop with the increasing of consolidation pressure.

It can be obtained from Figure 3 that (1) when the consolidation is small, the partition of primary-secondary consolidation can be easily got; however, the partition of primary-secondary consolidation becomes unclear with the increasing of consolidation load; (2) when the load ratio is the same, the *e*-lg*t* curves are no longer parallel to each other under the condition of *p* < 200 kPa, and this implied that the coefficient of secondary consolidation is not a constant and relates to the consolidation load. It also can be found from Figure 4 that the coefficient of secondary consolidation first increases and then starts to decrease with the increasing of consolidation pressure. However, when the *p* > 200 kPa, the coefficient of secondary consolidation approaches to a constant and does not change any more.

In summary, the correlation between coefficient of secondary consolidation and consolidation pressure depends on the consolidation state of soil. When the consolidation pressure is less than preconsolidation pressure, the soft soil is in an overconsolidated state and the coefficient of secondary consolidation increases with the increasing of consolidation pressure at this time. When the soft soil comes into a normally consolidated state as the value of consolidation load continue to increase, the coefficient of secondary consolidation will decrease with the increasing of consolidation pressure and approach to a constant, and the maximum is achieved as the consolidation pressure approaches the preconsolidation pressure. The results of one-dimensional consolidation tests of soft soil given by Nash et al. [11] and Yu et al. [12] also show the similar phenomena, and Yin et al. [13] consider that when the consolidation pressure is smaller than preconsolidation pressure, coefficient of secondary consolidation relates to the consolidation pressure. However, the test results of Bjerrum [14] and Shi et al. [15] also implied that the coefficient of secondary consolidation does not change with the consolidation pressure. This is because the sampling depth of soft soil is relatively shallow and the consolidation pressure given by tests is greater than preconsolidation pressure; in this condition, the soft soil is in the normally consolidated state and the coefficient of secondary consolidation does not depend on the consolidation pressure. Therefore, the formula (1) just can be used to calculate coefficient of secondary consolidation of normally consolidated soil

(1) $$\begin{array}{c} C_{a}=-\frac{\Delta e}{\text{lg}\left(t/t_{c}\right)}. \end{array}$$

Based on the above analysis, the influence of consolidation pressure on the secondary consolidation coefficient can be described by formula (2) which is according to research results established by Mesri and Castro [16]

(2) $$\begin{array}{c} C_{a}=\alpha C_{c}, \end{array}$$

where *C*_*c*_ is the coefficient of compressibility of soil which can be got from oedometer test, and, according to the test results, the relationship between coefficient of secondary consolidation and coefficient of compressibility of the soil studied in this paper can be written as *C*_*a*_ = 0.03*C*_*c*_.

### 3.2. Shear Creep

**Figure 5:**
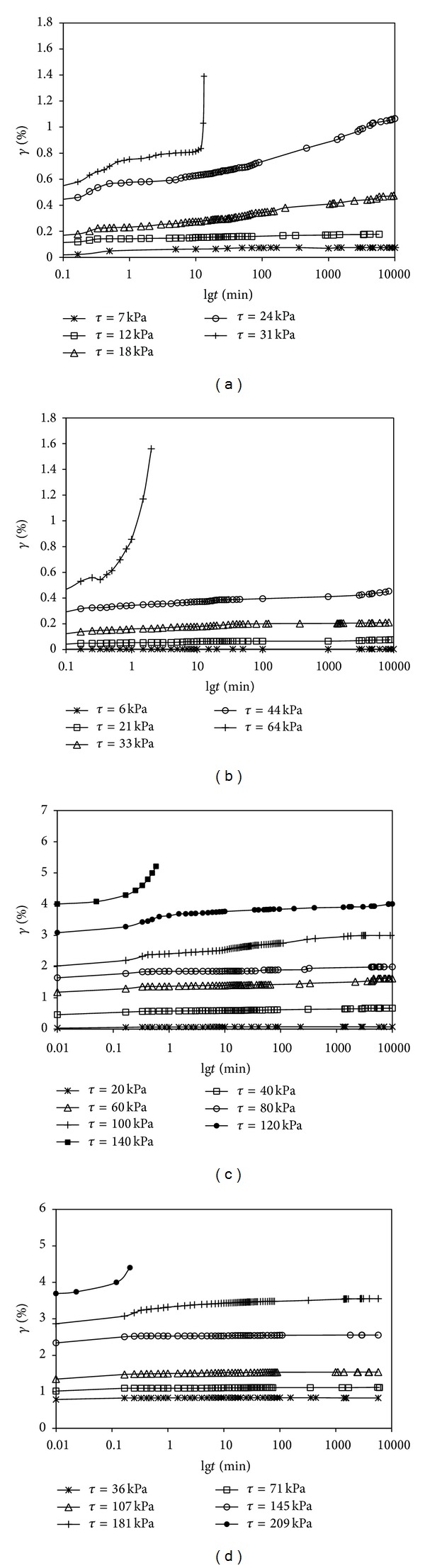
(a) Direct shear creep test for *p* = 50 kPa. (b) Direct shear creep test for *p* = 100 kPa. (c) Direct shear creep test for *p* = 200 kPa. (d) Direct shear creep test for *p* = 300 kPa.

The tests show that the shear creep curves under different consolidation pressures are roughly the same; however, when the consolidation pressure is small, the nonlinear relationship of the *γ* ~ lg*t* curves is more obvious. Variations of the shear strain *γ* with respect to time are plotted in Figures 5(a)–5(d) with different pressures.

It can be seen from Figures 5(a)–5(d) that the instantaneous deformation and shear strain rate increase with the increasing of shear stress under the same load. When the shear stress is small, such as *τ* = 0.2*τ*_*f*_~0.4*τ*_*f*_, the instantaneous deformation accounts for a large part of deformation, and the value of shear strain hardly changes with the increasing of time. In other words, there is almost no shear creep deformation under small shear stress. When the shear stress becomes more and more large, the shear creep deformation is more and more obvious and comprises much of displacement, which will cause more pronounced nonlinear relationship between shear strain and time. As long as the shear stress increases to a peak strength *τ*_*c*_, the soil sample will be damaged in a very short time (0.1~10 min) without clear accelerated creep before damaged, and failure time decreases with the raising of consolidation pressure. It indicates that the indirect sheer creep failure of soil is mainly controled by shear stress rather than the accumulation of shear strain. In addition, based on the results of shear creep tests under different consolidation pressures, we can find that *τ*_*c*_/*p* = 0.6~0.7; this is to say, consolidation effect can increase the peak strength of directly shear creep. When the shear stress attains the peak strength *τ*_*f*_ of consolidated-drained direct shear test, the soil sample will not have directly shear creep damage, however, the soil sample will be destroyed once the shear stress attains the strength *τ*_*c*_. We can find that *τ*_*c*_ is greater than *τ*_*f*_ under the same consolidation pressure, and *τ*_*c*_/*τ*_*f*_ grows bigger with the increasing of consolidation pressure.

**Figure 6:**
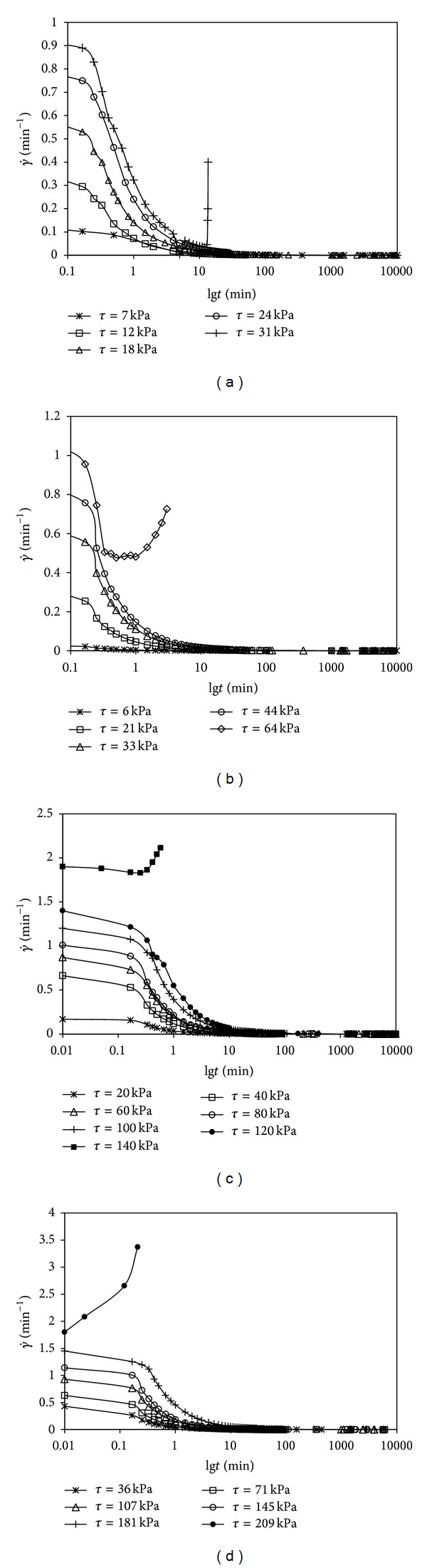
(a) $\dot{\gamma }$~lg*t* curves for *p* = 50 kPa. (b)$\;\dot{\gamma }$ ~lg*t* curves for *p* = 100 kPa. (c) $\dot{\gamma }$~lg*t* curves for *p* = 200 kPa. (d) $\dot{\gamma }$~lg*t* curves for *p* = 300 kPa.

Figures 6(a)–6(d) give curves of the average shear strain rate $\dot{\gamma }$ versus time under different pressures. It is found that the shear strain rate becomes bigger with the increasing of shear stress under a certain consolidation pressure, and, before shear stress reaches the peak strength *τ*_*c*_ of direct shear creep, the shear strain rate decreases with time at each level of shear stress.

At the beginning of loading, the shear strain rate reduces rapidly and then quickly approaches to zero with the development of time; however, in this case, the soil samples will not be damaged because of the large initial shear strain rate. Once the shear stress attains to *τ*_*c*_, the shear strain rate will increase sharply in a very short time after loading and finally lead to the failure of soil sample. With the increase of the consolidation pressure, the failure time decreases and the stage of decreasing of strain rate is getting shorter. It come to the conclusion that the initial high shear strain rate does not cause the shear creep damage of soil, while the upward development of shear strain rate will make the final destruction of soil.

### 3.3. Triaxial Creep

**Figure 7:**
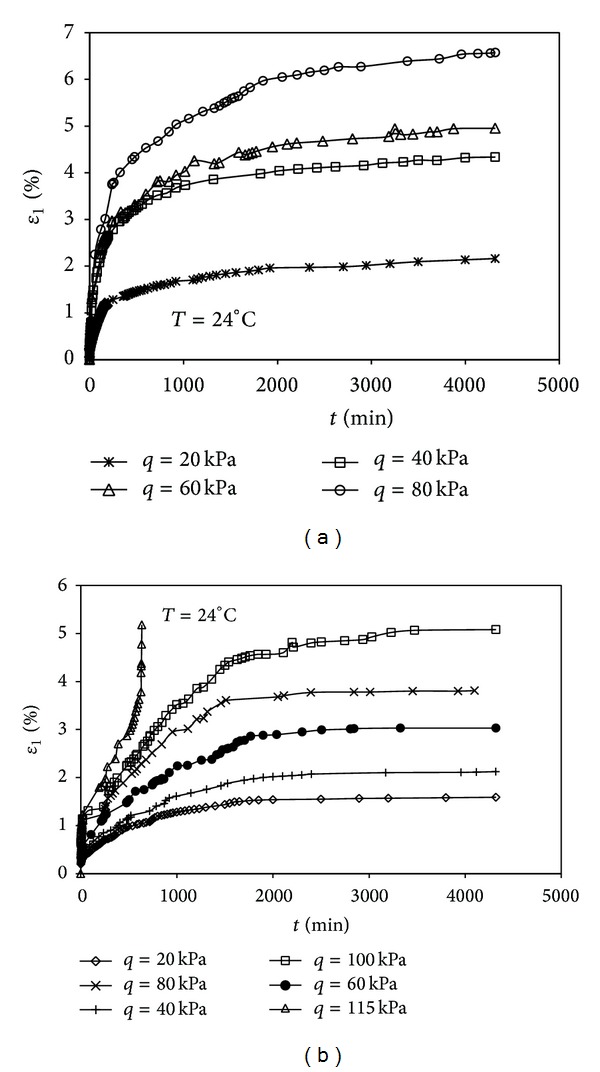
(a) Creep curve of drained creep test (*p* = 200 kPa). (b) Creep curve of undrained creep test (*p* = 200 kPa).

**Figure 8:**
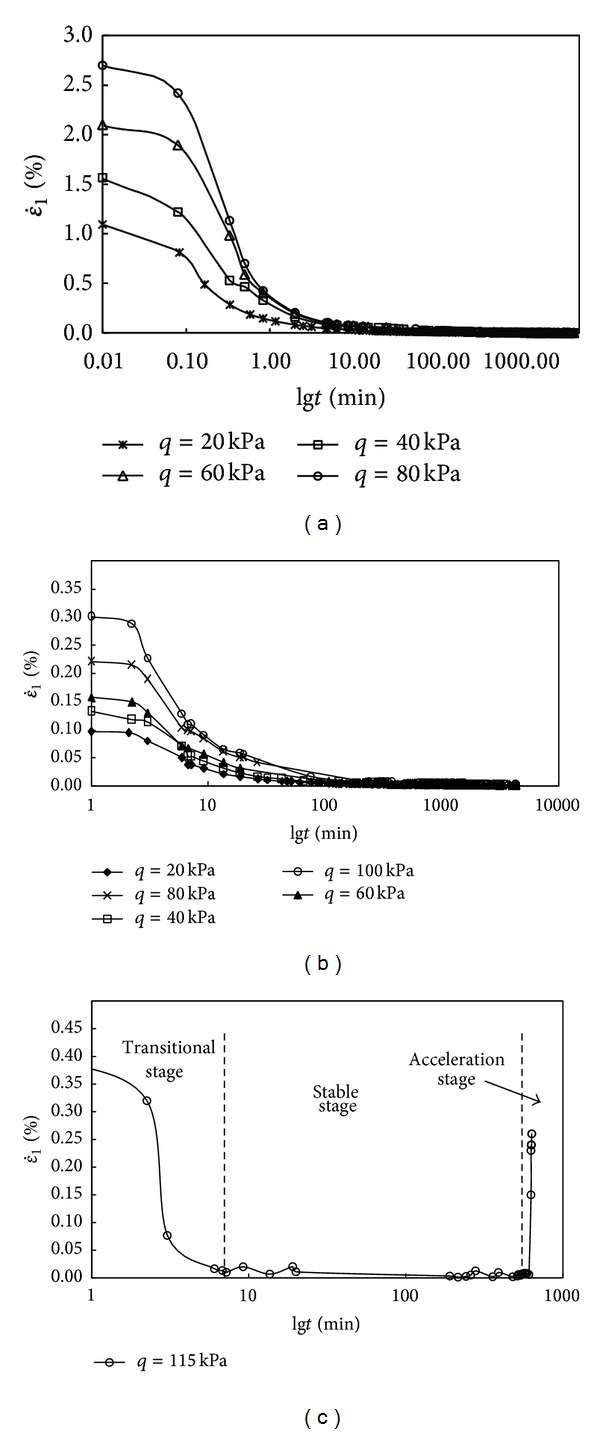
(a) ${\dot{\varepsilon }}_{1}$~*t* curve of drained creep test (*p* = 200 kPa). (b) ${\dot{\varepsilon }}_{1}$~*t* curve of undrained creep test (*p* = 200 kPa). (c) ${\dot{\varepsilon }}_{1}$~*t* curve of undrained creep test (*p* = 200 kPa).

The triaxial creep test results are shown in Figures 7 and 8, where *ε*_1_ is axial strain, and ${\dot{\varepsilon }}_{1}$ is axial strain rate. In order to get the axial strain-time curves of loading, respectively, the “Chen method” is used [17], and the curves of axial strain versus time under different deviatoric stresses, respectively, are plotted in Figures 7(a) and 7(b). The results show that the axial strains and creep characteristics of the soil samples are different due to the different drainage conditions, and the axial strain of undrained creep test is smaller than that of drained creep test under the same deviatoric stress. In the case of drained creep test, the soil sample has large deformation but no failure occurs; however, the displacement of soil sample increases sharply once the deviatoric stress attains 115 kPa in the undrained creep test. Under the condition of undrained creep, the deformation is caused just by the creep; however, in the drained creep test, the deformation is affected by the consolidation and creep together. Within the scope of a certain stress in both drained creep and undrained creep test, the soil sample will not be damaged because of accelerated creep, which is always in the state of steady creep with the increasing of deviatoric stress. In the undrained creep test, the creep effect is very small at lower stress level, but the creep effect of soil sample will become more and more obvious with the increasing of deviatoric stress, and, once the deviatoric stress reaches the value of 115 kPa, the soil sample will be damaged due to the accelerated creep after a period of steady creep. The result is not surprising since, in the drained creep test, the soil sample is consolidated, and the strain is composed of shear strain and volumetric strain; however, the soil sample is not consolidated in the undrained creep test, and there is just shear strain. Contrast with shear creep, there is steady creep before accelerated creep in triaxial undrained creep test because of the lack of lateral restraint and known failure surface, and the failure time is much longer in triaxial undrained creep test.

The relationships between strain rate and time under different deviatoric stresses are sketched in Figures 8(a)–8(c). It is found that, when the deviatoric stress is lower, the initial strain rate is maximum in spite of the drainage condition, but the soil sample will not be destroyed. The strain rate in the drained test is much bigger than strain rate in the undrained test. This is because the deformation of drained creep includes not only creep but consolidation, and the consolidation deformation is greater than creep deformation. From Figures 8(a) and 8(b), we can obtain that when the soil sample is in the condition of undrained and in the drained test which is at a state of certain deviatoric stress (*p* ≤ 100 kPa), the ${\dot{\varepsilon }}_{1}$ decreases as the time increases; especially at the beginning of loading, the $\dot{\varepsilon }$ reduces precipitously, and then the drop will slow down along with the development of time and approach to zero. From Figure 8(c), it is found that once the deviatoric reaches the value of 115 kPa in the case of undrained creep, the ${\dot{\varepsilon }}_{1}$ reduces with time firstly and then is kept stable over a period of time; however, when *t≈*580 min, the ${\dot{\varepsilon }}_{1}$ begins to increases sharply and brings about the final creep damage of soil sample, and the whole process can be divided into three stages as shown in Figure 8(c).

Therefore, though the triaxial creep test relates to shear stress, it is more affected by the consolidation state. When the soil sample is in the condition of drainage, the creep effect can be ignored, and the soil sample is extremely easily damaged by shear creep in the undrained test. However, the drainage condition of practical engineering always lies between drainage creep test and undrained creep test, so it is necessary to consider the effects of consolidation which also cause the study to be more complex.

## 4. Conclusions

In this paper, a series of tests are performed to study the creep characters of soft soil in interactive marine and terrestrial deposit of Pearl River Delta. And the following conclusions may be drawn.

1. The correlation between coefficient of secondary consolidation and consolidation pressure depends on the consolidation state of soil. Only when the soil is in the normal consolidation state, the coefficient of secondary consolidation can be approximately regarded as a constant. The formula *C*_*a*_ = *αC*_*c*_ established by Mesri is appropriate for the soil samples studied in this paper, where the value of *α* is 0.03.
2. Under consolidation, the indirect sheer creep failure of soil is mainly controlled by shear stress rather than the accumulation of shear strain, and, when the shear stress is small, there is almost no shear creep deformation. The peak strength of shear creep *τ*_*c*_ is greater than the peak strength of slow shear test *τ*_*f*_ under the same consolidation pressure, and *τ*_*c*_/*p≈*0.6~0.7.
3. In the triaxial creep tests, when the drainage conditions are different, the axial strains and creep characteristics of soil samples are different, and the axial strain of undrained creep test is smaller than the that of drained creep test.
4. Though the triaxial creep test relates to shear stress, it is more affected by the consolidation state. When the soil sample is in the condition of drainage, the creep effect can be ignored, and the soil sample is extremely easily damaged by shear creep in the undrained test.
5. In the filled project, we should control the loading rate to avoid the foundation failure caused by the fast loading rate. In some practical soft soil engineering, the excess pore water pressure of soil will continue to rise and the effective pressure will reduce even the load is constant, which is the result of soil creep. Therefore, the ground should be treated firstly when we construct the structure on soft soil ground to improve the drainage condition of soil, which can avoid the shear creep failure.

## Figures and Tables

**Table 1:** Peak strength of consolidated-drained direct shear test under different consolidation pressures.

Consolidation pressure *p*/(kPa)	50	100	200	300

Peak strength of consolidated-drained direct shear test *τ*_*f*_/(kPa)	29.4	54.6	100.4	147.8
